# The red thread between methylation and mutation in bacterial antibiotic resistance: How third-generation sequencing can help to unravel this relationship

**DOI:** 10.3389/fmicb.2022.957901

**Published:** 2022-09-15

**Authors:** Stella Papaleo, Alessandro Alvaro, Riccardo Nodari, Simona Panelli, Ibrahim Bitar, Francesco Comandatore

**Affiliations:** ^1^Romeo ed Enrica Invernizzi Pediatric Research Center, Department of Biomedical and Clinical Sciences, University of Milan, Milan, Italy; ^2^Romeo ed Enrica Invernizzi Pediatric Research Center, Department of Bioscience, University of Milan, Milan, Italy; ^3^Department of Microbiology, Faculty of Medicine and University Hospital in Pilsen, Charles University, Pilsen, Czechia; ^4^Biomedical Center, Faculty of Medicine, Charles University, Pilsen, Czechia

**Keywords:** adaptive antibiotic resistance, DNA methylation, mutations, third-generation sequencing, bacterial epigenetics, DNA repair systems

## Abstract

DNA methylation is an important mechanism involved in bacteria limiting foreign DNA acquisition, maintenance of mobile genetic elements, DNA mismatch repair, and gene expression. Changes in DNA methylation pattern are observed in bacteria under stress conditions, including exposure to antimicrobial compounds. These changes can result in transient and fast-appearing adaptive antibiotic resistance (AdR) phenotypes, e.g., strain overexpressing efflux pumps. DNA methylation can be related to DNA mutation rate, because it is involved in DNA mismatch repair systems and because methylated bases are well-known mutational hotspots. The AdR process can be the first important step in the selection of antibiotic-resistant strains, allowing the survival of the bacterial population until more efficient resistant mutants emerge. Epigenetic modifications can be investigated by third-generation sequencing platforms that allow us to simultaneously detect all the methylated bases along with the DNA sequencing. In this scenario, this sequencing technology enables the study of epigenetic modifications in link with antibiotic resistance and will help to investigate the relationship between methylation and mutation in the development of stable mechanisms of resistance.

## Antibiotic resistance

The discovery of antibiotic compounds has revolutionized the way in which we face infectious diseases: antibiotic drugs have saved several lives worldwide, fighting infections that nowadays seem easy to cure, but were not so simple in the past (Aminov, 2010; Ventola, 2015). However, the misuse of antibiotics led to the emergence and spread of antibiotic-resistant bacterial clones (Nature, 2013). Indeed, it is clear that the development of a novel antibiotic drug is often followed by the discovery of a novel resistance mechanism (Ventola, 2015).

### Genetic bases of antibiotic resistance

The word “antibiotic” defines the application of a compound used to kill or inhibit bacterial growth, more than a specific class of molecules (Waksman, 1947). Antibiotic drugs can be produced naturally by microorganisms or artificially through chemical synthesis. Each of these molecules affects a particular target and blocks a specific pathway, allowing classification in accordance with its mechanism of action: there are antibiotics targeting the cell wall (beta-lactams and glycopeptides), inhibitors of protein biosynthesis that block the 30S subunit (aminoglycosides and tetracyclines) or the 50S one (macrolides, chloramphenicol, oxazolidinones), inhibitors of DNA replication (quinolones), and folic acid metabolism inhibitors (Sulfonamides and trimethoprim) (Kapoor et al., 2017). Due to the differences between antibiotic mechanisms of action, the resistant strains withstand antibiotic treatment in several ways. The main resistance mechanisms can be summarized in three ways to face antibiotic attack: mutating the target site of the antibiotic or modifying it with the help of specific enzymes (Lambert, 2005; Schaenzer and Wright, 2020), inducing a modification of the antibiotic, also operated by specific enzymes (Ramirez and Tolmasky, 2010; Wilson, 2014), or increasing the efflux pump activity, which transport the drug out of the cell (Webber and Piddock, 2003).

Thus, it seems clear that a resistant strain needs a genetic determinant to overcome antibiotic attack: resistance can originate from mutations or it could be acquired through horizontal gene transfer of mobile genetic elements (Windels et al., 2020). Most of the Gram-positive organisms have a single resistant trait, making resistance detection through molecular investigation quite accurate. The situation is different for Gram-negative organisms, where antibiotic resistance is more heterogeneous: identifying it from the molecular analysis is tough and the absence of a resistance gene doesn't always mean that the strain is susceptible to a specific antibiotic (Yee et al., 2021).

Furthermore, adaptive antibiotic resistances (AdR) have been observed: resistance phenotypes not clearly associated with specific genetic determinants, such as mutations or gene presence/absence.

### AdR: Adaptive antibiotic resistance

**Figure 1:**
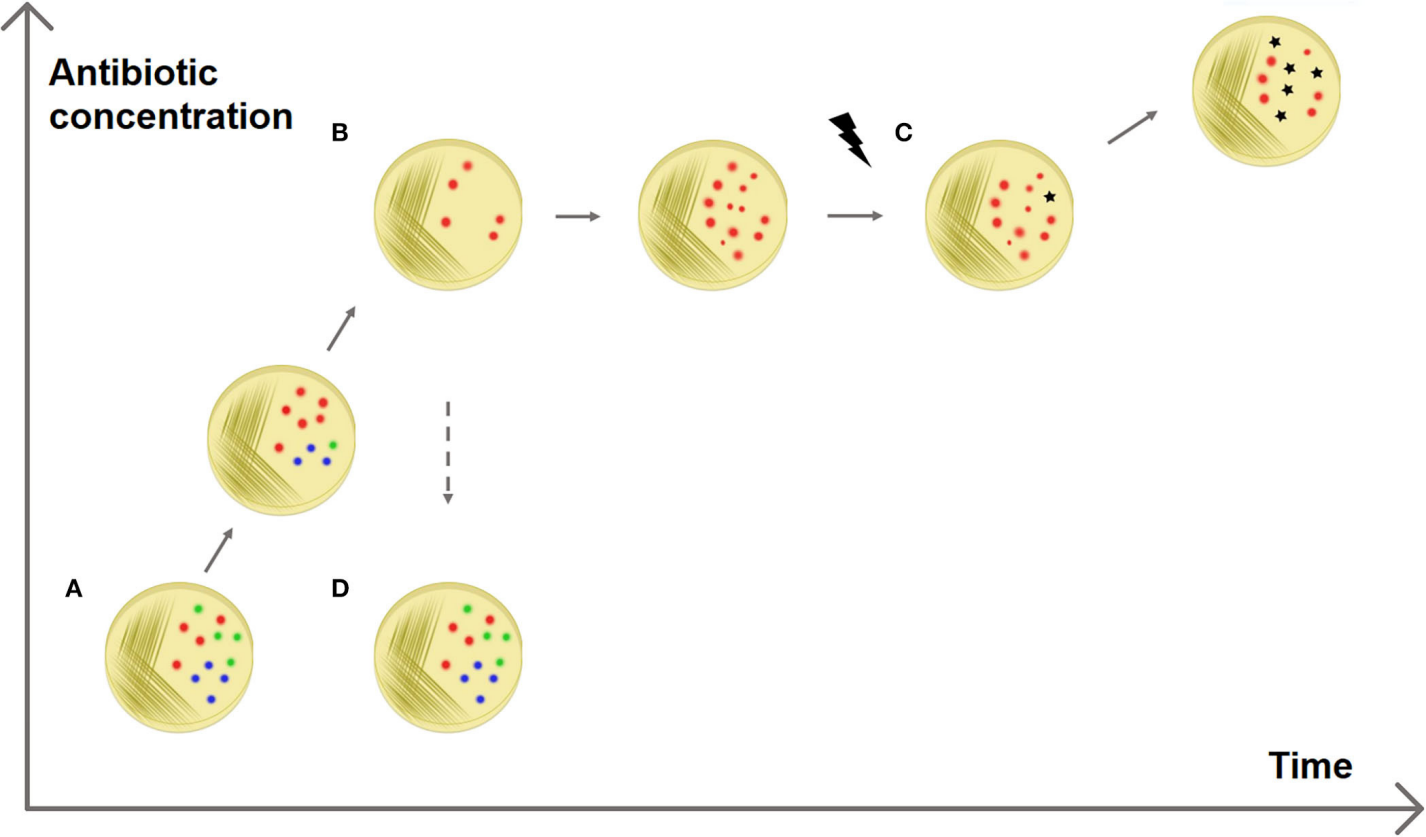
Adaptive antibiotic resistance. The figure shows a possible explanation for the selection of resistances. **(A)** A sensitive bacterial population has a heterogeneous methylation pattern (represented by red, blue, and green dots). **(B)** It has been observed that when the population is exposed to increasing antibiotic concentrations some members survive better than others, without the presence of resistance genes (Motta et al., 2015). This can be explained with epigenetics: it is possible that a specific methylation pattern (red dots) increases the survival rate, e.g., enhancing the expression of efflux pump genes. **(C)** This fast epigenetic-based resistance mechanism allows the population to survive antibiotic exposure for enough time that resistant mutated strains can be selected. **(D)** AdR is a transient resistance: if the antibiotic is removed, the resistant methylation pattern is no longer advantageous and the population returns heterogeneous (and sensitive).

AdR is defined as the ability of the strain to temporarily withstand antibiotic presence, achieved by changes in gene or protein expression (Fernández and Hancock, 2013). It can emerge when a strain is subjected to sub-inhibitory increasing antibiotic concentrations for a small amount of time (George and Levy, 1983; Barclay et al., 1992; Toprak et al., 2011). The AdR phenotype appears quickly and is reversible, although it is inheritable and transmissible to subsequent generations. Once the treatment with the antibiotic stops, the resistant cells revert to the susceptible phenotype, as opposed to intrinsic or acquired resistance, which is stable among the generations (Fernández and Hancock, 2013) (Figure 1).

In this regard, Adam et al. (2008) investigated the emergence of antibiotic resistance in *Escherichia coli* when exposed to low concentrations of antibiotics and they found out that the different gene expression patterns were the basis of the development of AdR. The authors excluded the possibility of a mutation-mediated resistance for three reasons: (i) the survival rates were too high; (ii) the resistance MIC increased with the antibiotic concentration; (iii) the reverting frequency after antibiotic removal (around 50%) was too high for a genetic-mediated mechanism (i.e. mutation or resistance genes). These results and the investigation of differential gene expression on resistant strains led the authors to propose that the observed resistance phenotype was due to epigenetic regulation (Adam et al., 2008).

## Bacterial epigenetics

The term “Epigenetics” was coined by Conrad Waddington to define the branch of biology that aimed to unravel the mechanisms under the development of a specific phenotype given a certain genotype (Goldberg et al., 2007). Epigenetic modifications are stable, inheritable, and reversible modifications of the DNA or of the histones, among which the most known and studied is methylation. This phenomenon was firstly investigated in eukaryotic organisms, where methylation of the cytosines (5-methylcytosine) in particular regions of the genome, CpG islands, was found to be involved in gene expression regulation (Moore et al., 2013). The first studies on bacterial epigenetics were published in 1955, when the presence of N6-Methyladenosine base was discovered in *E. coli* (Dunn and Smith, 1955).

In eukaryotic genomes, it is possible to observe two different methylated bases: 5-Methylcytosine and N6-Methyladenosine. Bacterial genomes can instead harbor N6-Methyladenosine, 5-Methylcytosine, and 4-Methylcytosine. The most-represented modified base in bacteria is N6-Methyladenosine, and 4-Methylcytosine is exclusive to bacteria and archaea (Sánchez-Romero and Casadesús, 2020) (Supplementary Table S2).

Another mechanism of bacterial epigenomics, whose importance is rising nowadays, is phosphorothioation, which is the first physiological modification of the DNA backbone to be discovered (Wang et al., 2007) (Supplementary Table S2).

### DNA methylation

DNA base methylation is operated by a class of enzymes called DNA methyltransferases, able to catalyze the addition of a methyl group to a DNA base from a donor molecule, e.g., the S-adenosylmethionine (SAM) (Cheng, 1995). The methyltransferases recognize and methylate specific motifs on the DNA double strand. This reaction takes place immediately after the replication, when the DNA is hemimethylated: the parental strand is methylated and the daughter strand is not. The methyltransferases recognize the already-methylated motif on the parental strand and methylate the corresponding position on the other strand (Adhikari and Curtis, 2016). Methyltransferases are not error-free and they can fail to correctly recognize and methylate their motifs. It can happen for different reasons: randomly, for steric hindrance (Casadesús and Low, 2006) or for reduced processivity due to sequences flanking the recognition motifs (Peterson and Reich, 2006).

When the methyltransferase fails to methylate a motif, it produces a hemimethylated position. This can cause the unmethylation of that site in the daughter cells, after DNA replication. This effect occurs because during DNA replication methyltransferases use each strand as a stamp for the methylation of the newly synthesized DNA daughter strands. Thus, the motif on the methylated strand will be inherited as methylated, while the other as unmethylated (Sánchez-Romero and Casadesús, 2021). This phenomenon can generate bacterial populations genetically identical but with heterogeneous methylation patterns.

Methyltransferases are mainly classified into two classes: those coupled to cognate restriction endonucleases (as part of Restriction-Modification, RM, systems) and the other lacking these paired enzymes (called orphan methyltransferases) (Adhikari and Curtis, 2016).

### Restriction-modification systems

**Figure 2:**
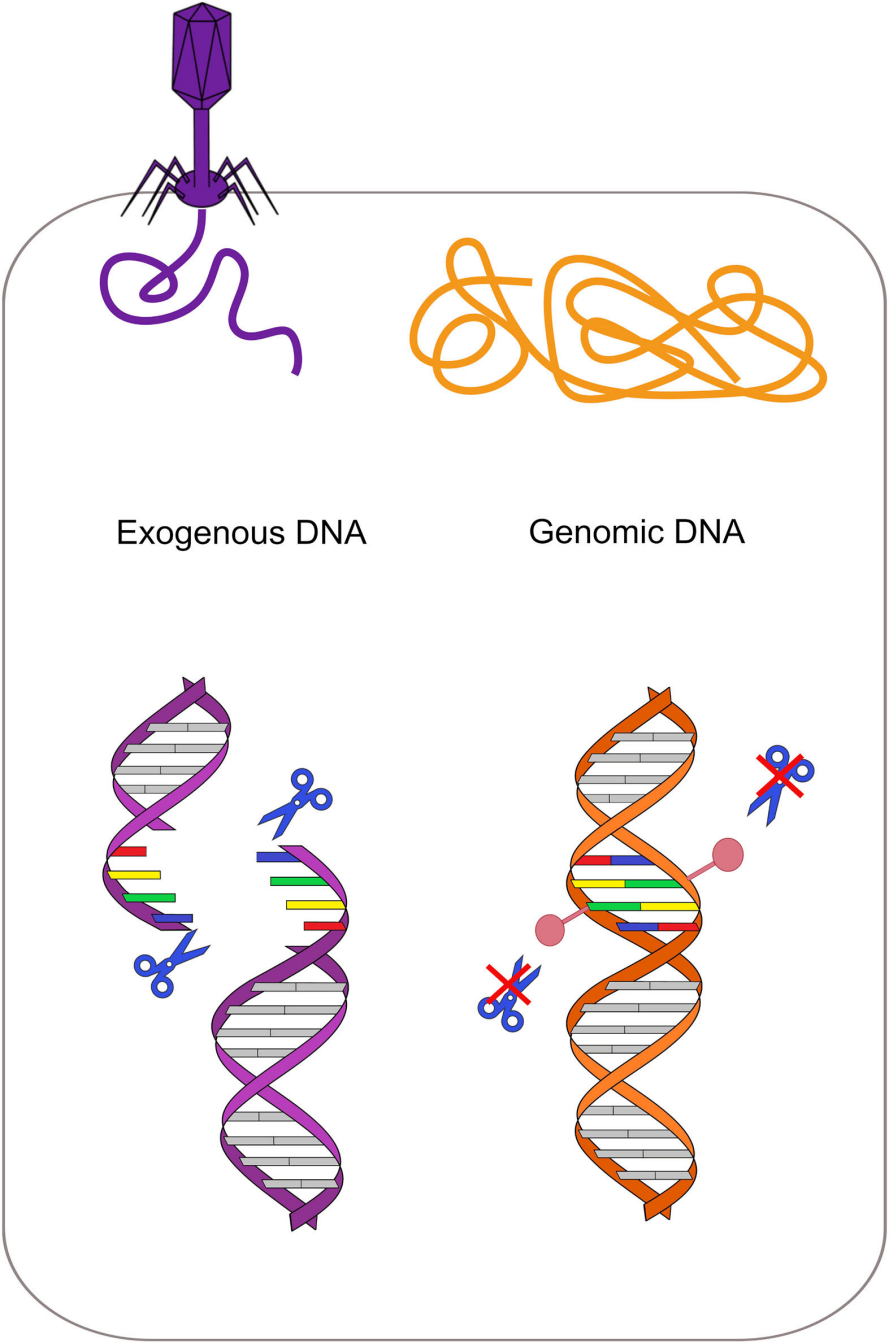
Restriction-modification system protects from exogenous DNA. Each restriction enzyme recognizes a specific motif and cleaves it if it is not methylated. In the bacterial genome (orange on the right), this specific motif is methylated by the cognate methyltransferase, while the same motif is unmethylated in the phage DNA (violet on the left). Restriction enzymes can thus cleave the phage DNA only, protecting the bacterium.

RM systems involve a methyltransferase that recognizes and methylates a particular motif, and a cognate restriction endonuclease that cleaves the same motif if it is unmethylated (Figure 2) (Vasu and Nagaraja, 2013). The main role of bacterial RM systems is to protect them from exogenous DNA, as a sort of primitive immune system (Jensen et al., 2019). However, recent studies revealed that methyltransferases can be also involved in transcriptional regulation (Vasu and Nagaraja, 2013).

The role of RM systems in genome protection makes them a driver for the acquisition and maintenance of mobile genetic elements (Spadar et al., 2021), directly affecting the transformation efficiency (Nye et al., 2019).

There are bacterial plasmids that, exploiting this system, increase their spreading capability. For instance, experimental evidence shows that the IncA/C plasmid, which encodes three methyltransferases genes, is able to increase its conjugation success rate in *Vibrio cholerae* modifying the bacterium methylation pattern. Indeed, the silencing of these three methyltransferases genes blocks the ability to transfer the plasmid among *Vibrio cholerae* strains and from *Vibrio cholerae* to *Escherichia coli* (Wang et al., 2019).

**Figure 3:**
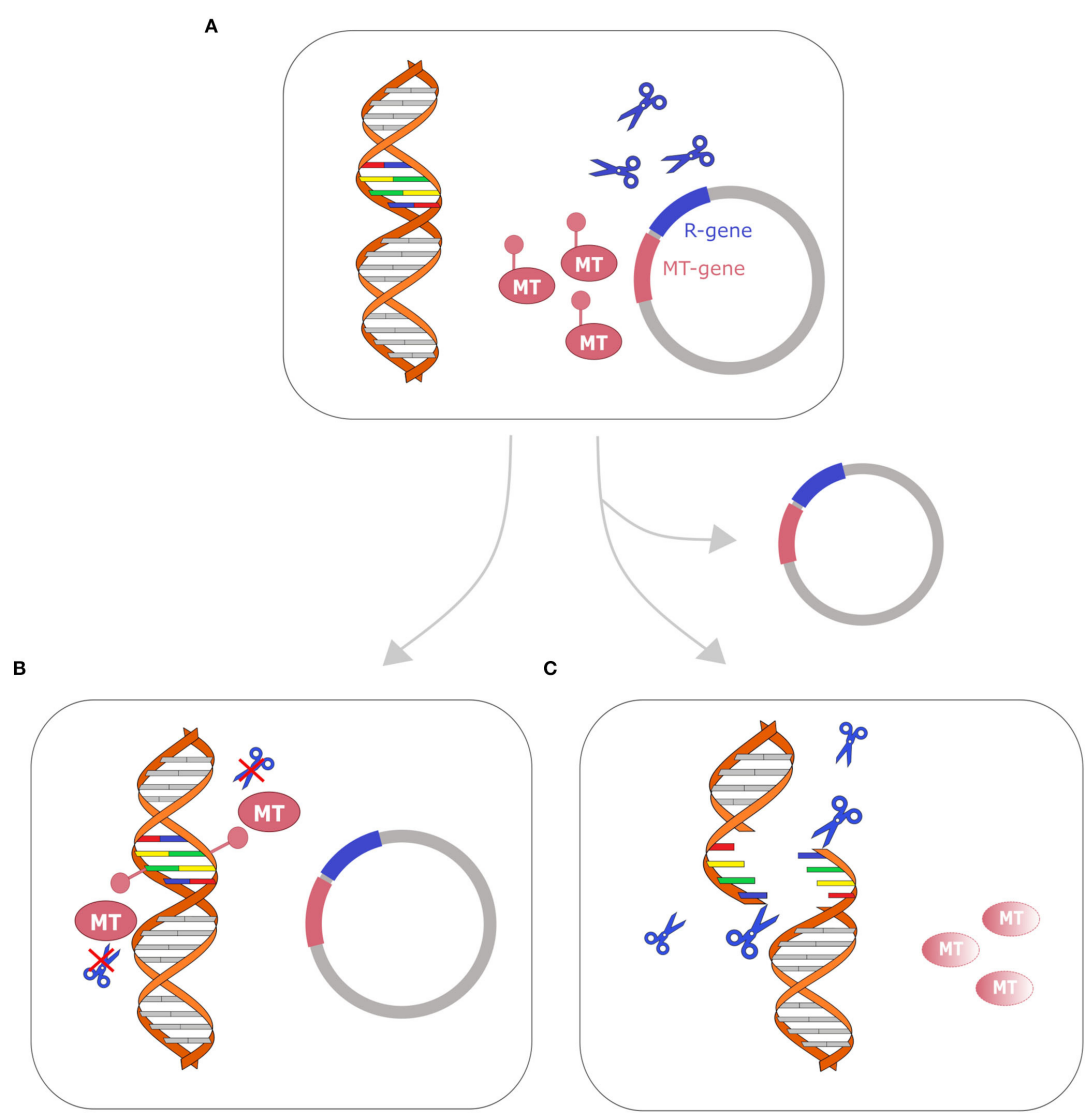
Restriction-modification system as a toxin–antitoxin system. Cell with plasmid that encodes R-M genes toxin–antitoxin system is represented on the top **(A)**. After replication, two fates are possible: **(B)** if the cell retains the plasmid (bottom left), the genomic DNA is methylated by the methyltransferase (MT) and the restriction enzyme (represented with blue scissors) cannot cleave the DNA; **(C)** if the cell loses the plasmid, the methyltransferase (MT) is fastly degraded, while the restriction enzyme remains active and cleaves the DNA, leading to cellular death. A similar mechanism has been described in several toxin–antitoxin systems (Kulakauskas et al., 1995; Unterholzner et al., 2013; Mruk and Kobayashi, 2014).

RM systems are involved in plasmid maintenance as they work like toxin–antitoxin (TA) systems (Kulakauskas et al., 1995). TA systems are usually composed of two proteins: a stable and lethal toxin and an unstable antitoxin, which binds and stabilizes the toxin, making it harmless. When this couple of enzymes is located on a plasmid, bacterial cells that lose that plasmid will die. Indeed, immediately after the plasmid loss, the toxin is still active while the antitoxin is rapidly degraded. This mechanism makes the plasmid indispensable for bacterial cell survival (Unterholzner et al., 2013). When the genes of methyltransferase and its cognate endonuclease are localized on the same plasmid, the functioning of the RM system can recall a toxin–antitoxin system. The restriction endonuclease acts as a toxin while the methyltransferase acts as an antitoxin, protecting the chromosomal DNA from cleavage. Indeed, plasmid-free cells can't methylate anymore the target DNA, but they still have their counterpart restriction enzyme that cleaves the target DNA and leads to cellular death. This system, leading to postsegregational killing of plasmid-free cells, increases plasmid stability (Mruk and Kobayashi, 2014) (Figure 3).

### Orphan methyltransferases

**Figure 4:**
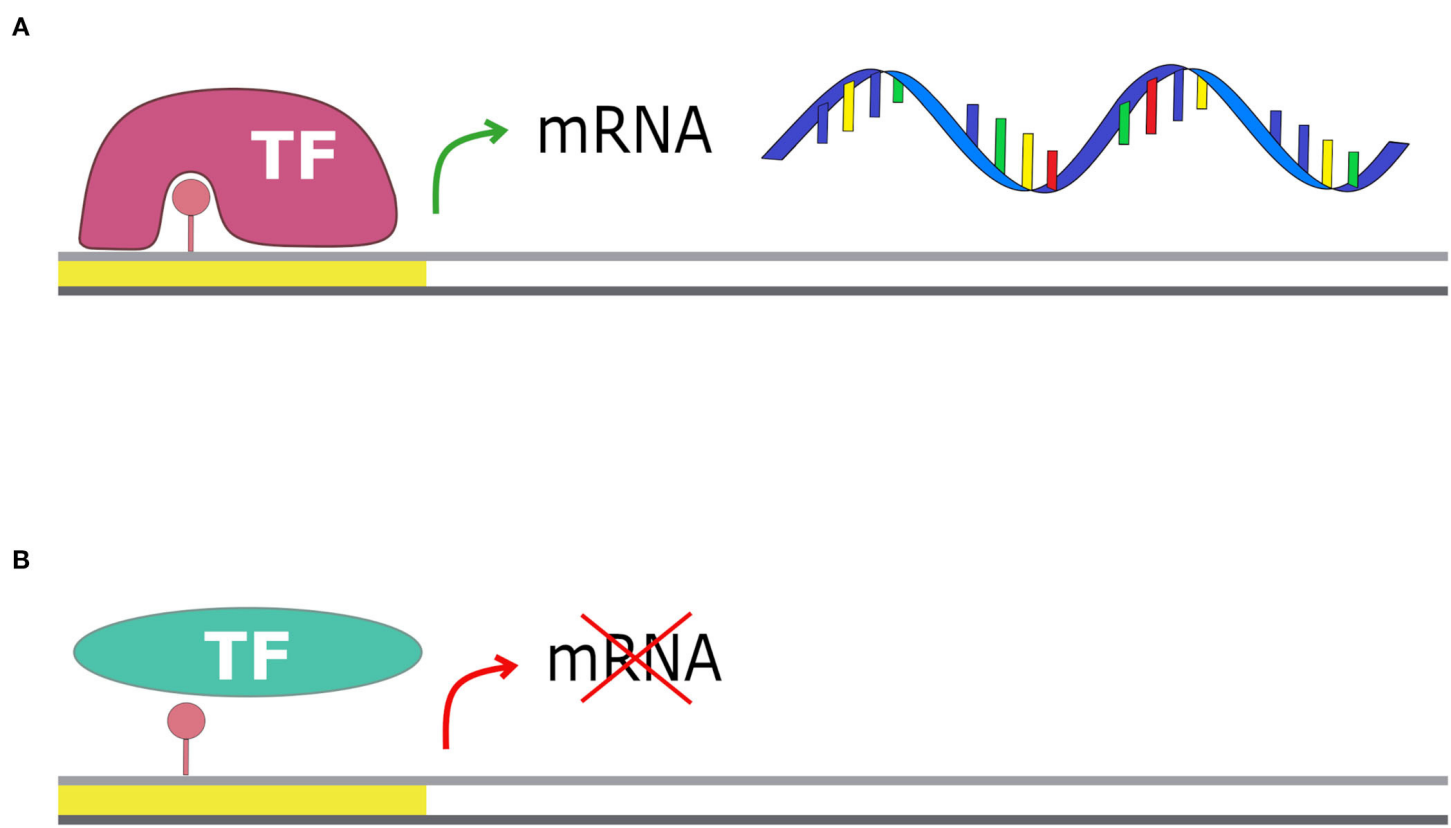
Methylation and transcription. The presence of a methyl group on the promoter region can affect gene transcription: **(A)** if the transcriptional factor has more affinity for the methylated motif, the transcription is enhanced; **(B)** if the transcriptional factor has less affinity for the methylated motif, it cannot bind the promoter and transcription is blocked.

Orphan methyltransferases are a group of methyltransferases highly conserved among Bacteria that lack the restriction part (Oliveira and Fang, 2021). These enzymes are involved in cellular processes such as the initiation of DNA replication, DNA repair mechanisms, and gene expression regulation (Adhikari and Curtis, 2016). The most investigated methyltransferase is deoxyadenosine methyltransferase (Dam) in *E. coli* (Schlagman et al., 1986; Messer and Noyer-Weidner, 1988; Palmer and Marinus, 1994; Calmann and Marinus, 2003). This enzyme is conserved in most of the Gammaproteobacteria, and it is an orphan adenine methyltransferase that targets the adenine of the palindromic motif 5′-GATC-3′ (Ghosh et al., 2020). The GATC motif is involved in the control of DNA replication initiation. Indeed, until the genomic DNA replication starts, the regulatory protein SeqA binds several of the GATC motifs present in the OriC locus, preventing the starting of the replication phase. DNA replication can then start only when SeqA is removed and all the GATC motifs in the OriC locus are fully methylated by Dam (Kang et al., 1999). Orphan methyltransferases are also involved in the regulation of gene expression: methylation of particular motifs can enhance or inhibit the binding of regulatory proteins, such as transcriptional activators or repressors, therefore, influencing gene expression (Figure 4) (Oshima et al., 2002). Other effects of methylation on DNA are the changing of DNA curvature, the reduction of DNA thermostability, and the competition with DNA-binding proteins (Marinus and Casadesus, 2009).

### Phosphorothioation

Phosphorothioation (PT) is a chemical modification of the DNA that occurs not on bases, like methylation, but on DNA backbone: it is the substitution of a non-bridging oxygen on the phosphodiester bond with a sulfur atom (Wang et al., 2007). This oxygen–sulfur exchange is catalyzed by the cooperation of the products of the *dnd* gene cluster (*dnd*ABCDE) that involve a cysteine desulfurase (DndA), an iron–sulfur cluster protein (DndC), a protein with ATPase activity (DndD), a protein that binds nicked dsDNA (DndE) and DndB, that seems to be not essential for PT modification (Jian et al., 2021). This modification occurs on 5′GAAT3′/5′GTTC3′/5′GATC3′ and similar motifs (Wu et al., 2020). Phosphorothioation is linked with some important functions: (i) it confers protection to DNA against oxidative damage; (ii) it is often coupled with *dnd*FGHI genes, which cleave unmodified DNA motifs (likewise RM systems) (Tong et al., 2018); (iii) influence or inhibit restriction enzymes that cleave close to modified sites; (iv) it changes the affinity of regulatory proteins affecting gene expression regulation (Jian et al., 2021). Interestingly, both PT modification and DNA methylation systems can recognize the 5′-GATC-3′ motif, and thus a hybrid 5′-GPS6mATC-3′ can be produced (Chen et al., 2017).

## AdR as a bridge to stable antibiotic resistances

AdR has the potential to be the first line of defense against antibiotic exposure, unlike intrinsic and acquired resistance, which are achieved slowly by mutation or acquisition of resistance genes. This fast-appearing resistance is transient, and the resistant phenotype is easily reverted by the removal of the inducing condition. However, exploring possibilities in the survival scenario through the stochastic creation of different epigenetic lineages can be a fast way to withstand the antibiotic presence while searching for a more stable mechanism of resistance.

### Adapting mechanisms: Non-conventional methods of resistance

Epigenetic adaptive resistance is mainly related to changes in gene expression regulation by the enhancement or inhibition of certain cellular features (Supplementary Table S3). For instance, epigenetics regulation can lead to the enhancement of the expression of efflux pumps belonging to the resistance–nodulation–division (RND) superfamily able to extrude a wide range of toxins and antibiotics outside the cell (Motta et al., 2015). In fact, different expression levels of efflux pump genes such as *acrD, marR, rpoS, fabI*, and *lrhA* in adaptive antibiotic-resistant strains are related to Dam methylation (Hughes et al., 2021). The overproduction of these MDR efflux pumps is also related to a decreased permeability of the cell, controlled by the underexpression of membrane porins, such as OmpC, that reduce the intake of toxic compounds (Sánchez-Romero and Casadesús, 2014).

Another AdR mechanism concerns chaperonins, enzymes involved in protein folding (Beissinger and Buchner, 1998). It is known that resistance to antibiotics that interfere with translation can emerge by the overexpression of chaperonins (Carvalho et al., 2021). Indeed, the overexpression of chaperonins GroEL/GroES expand the mutational space, because they guarantee the correct folding of proteins despite the presence of potentially lethal mutations (Goltermann et al., 2015). There is experimental evidence that chaperonins are regulated by methylation in *Vibrio cholerae*: the deletion of the orphan methyltransferase *vchM* (5′-RCCGGY-3′ motif) is associated with groESL-2 upregulation in *V. cholerae* and a greater survival rate under aminoglycoside stress. The same effect has been observed in wt *V. cholerae* strains, when the 5′-RCCGGY-3′ motifs in the groESL-2 region are unmethylated (Carvalho et al., 2021). Antibiotics affect main bacterial processes, depending on the mechanisms of action. For this reason, they create an imbalance in normal cellular functions and alter the cellular redox state. Therefore, the lethality of the antibiotics is also linked to the oxidative stress that they induce in the cell (Dwyer et al., 2014). As stated above, phosphorothioation modification has both redox and nucleophilic properties that are likely to have effects on bacterial fitness in stressful environments (Kellner et al., 2017) such as antibiotic treatment.

### From fast and transient mechanisms (methylation) to slow and stable ones (mutation)

When a sensitive bacterium is exposed to an antibiotic molecule, the first-line reaction can be a fast modulation of gene expression by epigenetic mechanisms. During the time period of this epigenetic-based reaction, resistant strains could emerge and a novel antibiotic-resistant bacterial population can be selected (Olofsson and Cars, 2007).

For instance, antibiotic exposure can lead to a higher expression of genes associated with efflux pumps (Fernández and Hancock, 2013). This transient phenotype can be stabilized by acquiring DNA mutations that increase the efficiency and specificity of efflux mechanisms (Sandoval-Motta and Aldana, 2016).

In this scenario, there is a switch from a Fast and Transient Mechanism (FTM), where phenotypic changes are still reversible, to a Slow and Stable Mechanism (SSM) (Sandoval-Motta and Aldana, 2016), which may overcome the transient resistance with compensatory modifications (El'Garch et al., 2007), giving a permanent resistance against specific antibiotics.

Moreover, DNA methylation affects the mutation rate, thus one of the mechanisms behind the first fast response to the antibiotics may be the one that promotes a more stable adaptation (Sandoval-Motta and Aldana, 2016).

### How methylation is linked to mutation?

Bacteria can exploit adapting resistance mechanisms at the expense of other cellular functions.

**Figure 5:**
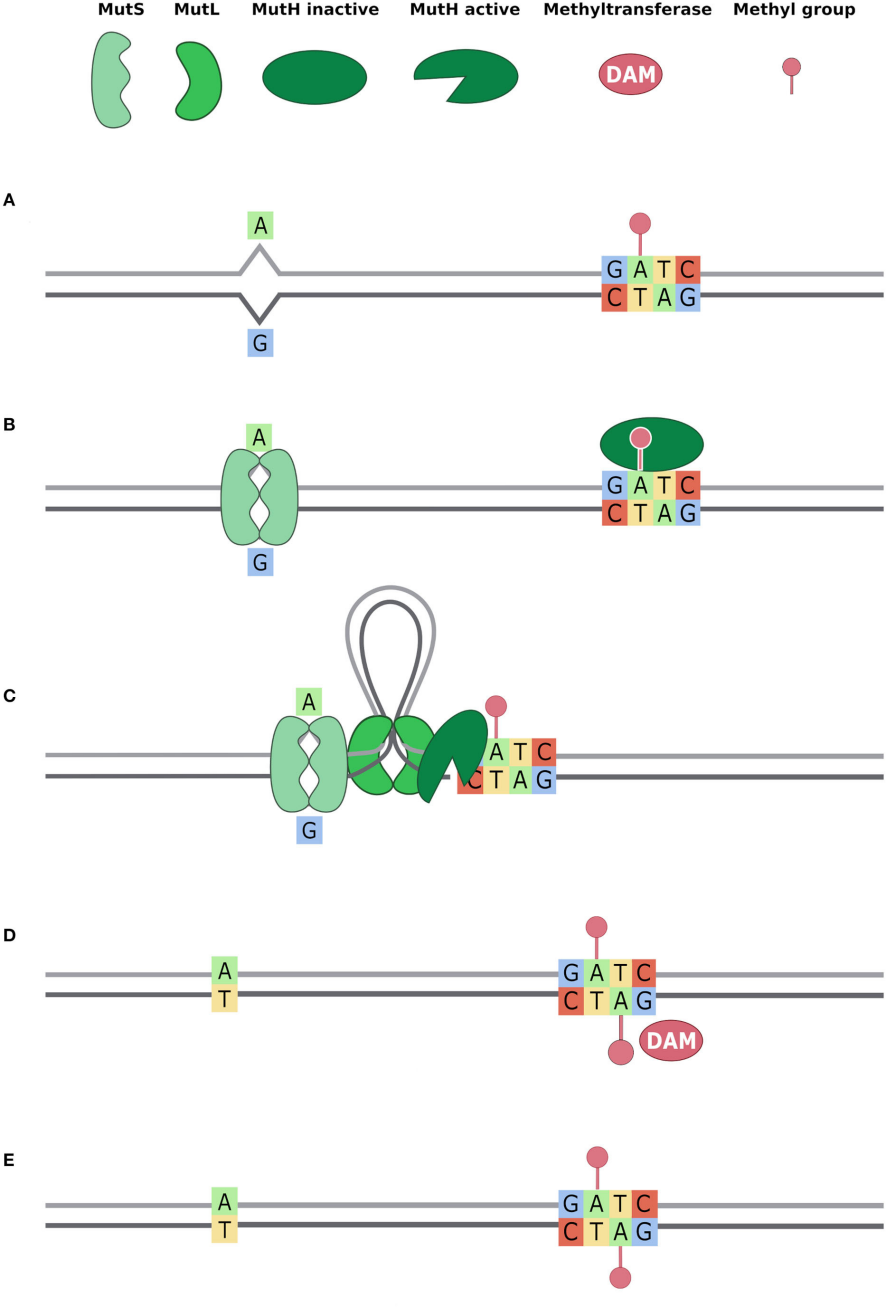
Methyl Mismatch Repair system (MMR) (also known as MutSLH). Dam methyltransferase methylates the GATC motifs right after the action of DNA polymerase and DNA repair systems (Acharya et al., 2003; Li, 2008; Sandoval-Motta and Aldana, 2016). Thus, when DNA repair systems bind the DNA, GATC motifs are methylated on the parental strand only. This allows the DNA repair system to distinguish, in a mismatch, the original base from the mutated one. In the figure, we describe the activity of MutSLH system in five steps **(A–E)**: **(A)** a T -> G mutation is represented and, downstream the mismatch, the nearest GATC site is methylated only on the parental strand; **(B)** the MutS dimer binds the mismatch and MutH binds hemimethylated GATC; **(C)** MutL dimer binds MutS dimer and forms a loop in the DNA, searching for the nearest GATC site to bind MutH. This activates MutH endonuclease activity that creates a nick in the unmethylated newly synthetised strand; **(D)** MutSLH complex recruits DNA helicase, exonuclease, DNA Pol III, and DNA ligase to fix the mismatch. Once the mismatch is fixed, Dam enzyme can methylate the GATC motif also on the other strand; **(E)** Both strands are methylated on the GATC site.

**Figure 6:**
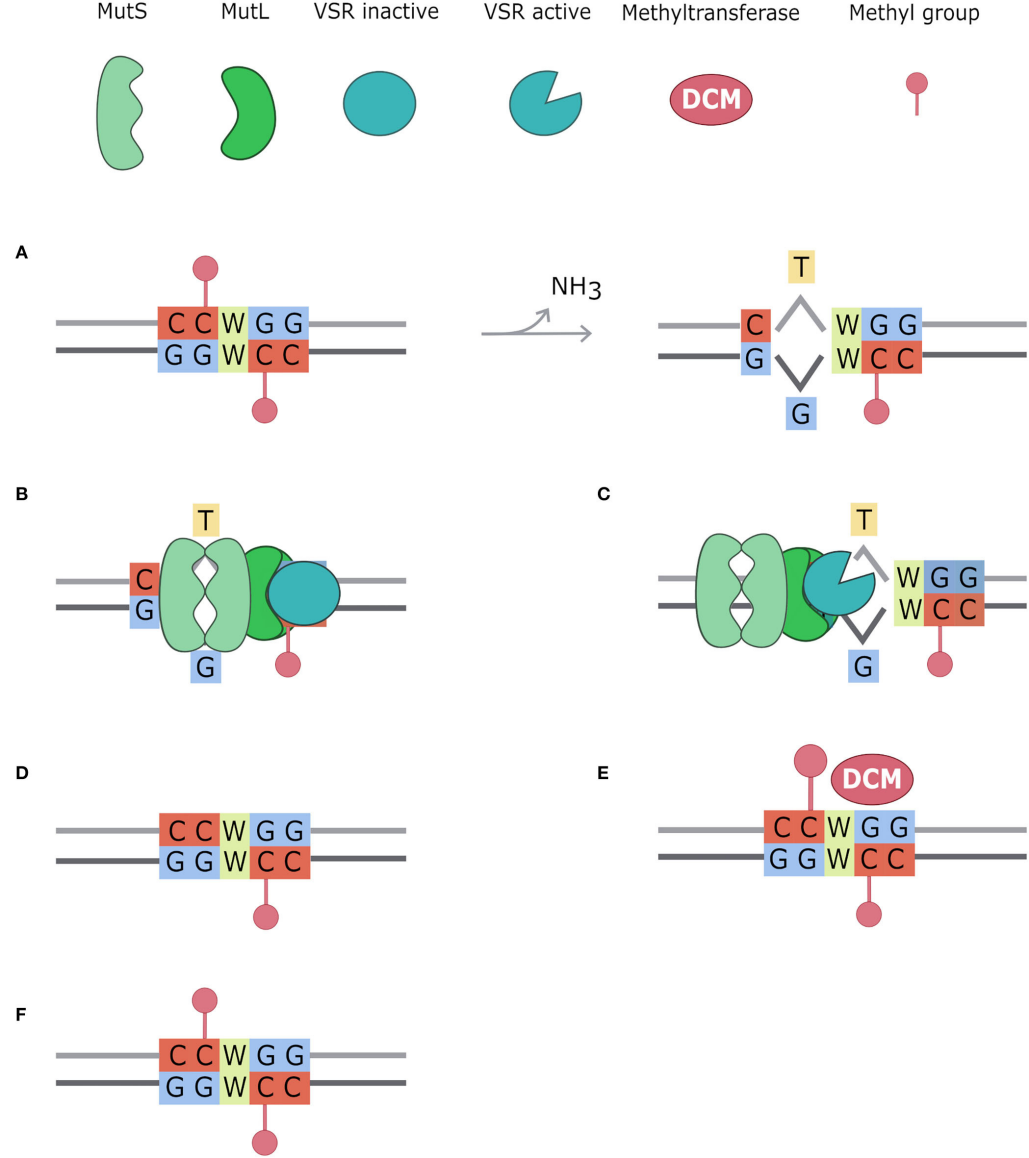
Very Short Patch repair system (VSP). Methylated cytosines spontaneously deaminate to Thymine creating T/G mismatches. The very short patch repair system (VSP) acts repairing these mismatches, always in favor of the Guanine (Drotschmann et al., 1998; Marinus, 2012). In this figure, the VSP system mechanism is divided into six steps: **(A)** CCWGG motifs are methylated on both strands by the Dcm enzyme. Methylated cytosines spontaneously deaminate to thymine, creating a mismatch with guanine; **(B)** MutS dimer recognizes the mismatch and binds MutL dimer which acts as a bridge with VSR protein; **(C)** VSR is activated by the MutSL complex and creates a nick in the strand containing the incorrect base, using as parental stand that containing the Guanosine; **(D)** the MutSL-VSR complex recruits DNA helicase, exonuclease, DNA polymerase, and DNA ligase to fix the mismatch; **(E)** CCWGG motif is restored and thus DCM methyltransferase can methylate it; **(F)** CCWGG is methylated on both strands.

Directing energies toward a high expression level of efflux pumps has a cost that bacteria pay with decreased fitness (Andersson and Levin, 1999; Ebbensgaard et al., 2020). For instance, it has been demonstrated that *E. coli* with high levels of efflux pumps show decreased activity in mismatch repair systems (Foster, 2005; El Meouche and Dunlop, 2018). Bacteria under stress conditions have a less functioning repairing system, such as for lower expression of *mut*S gene (El Meouche and Dunlop, 2018). MutS participates in two of the main DNA repair systems, in *E. coli* and other bacteria (Lieb et al., 2001): the methyl mismatch repair system (MMR) and the very short patch (VSP) repair system. The first one, also called the MutSLH pathway, is active during the exponential growth phase, in which it repairs replication errors (Figure 5). It is composed of MutS that recognizes mismatches and binds MutL, which recruits the endonuclease MutH. Cooperatively with other proteins, they restore the correct nucleotide sequence, repairing mismatch bases on the newly synthesized strand (Acharya et al., 2003). After DNA replication, there is a phase in which methylated Guanine, Adenine, Thymine and Cytosine (GATC) sites (with the adenine methylated to 6 mA) are present on the parental strand only because the Dam still has to methylate the newly synthesized strand. MutSLH is particularly active in this phase: after the MutS mismatch recognition, MutH binds the hemimethylated GATC sites and discriminates the parental strand from the newly synthesized strand for the presence of the methylated adenine (6 mA). Mismatch is thus repaired using the parental strand as a template and replacing the incorrect base on the newly synthesized one (Li, 2008; Sandoval-Motta and Aldana, 2016). When MutS is down expressed, this system can't work properly, therefore leading to a high mutation rate due to uncorrected mismatched bases caused by DNA polymerase errors (El Meouche and Dunlop, 2018). MutS is also involved in the VSP mismatch repair system, which fixes T/G mismatches in non-dividing cells (Figure 6). Indeed, methylated cytosines spontaneously deaminate at thymines, creating T/G mismatches. The VSP system is able to recognize and repair these mismatches with the cooperative work of MutS, which binds the mismatch, of the VSR endonuclease, which cuts the DNA, and MutL, which recruits the helicase (Drotschmann et al., 1998). VSP prevents the fixation of the mutation due to methylated cytosine deamination that results in a C to T transition. VSP works on the 5′-CCWGG-3′ motif (Marinus, 2012), where the inner cytosine is methylated by Dcm, an orphan methyltransferase. Thus, if MutS is lacking, VSP can't restore T/G mismatches. In this scenario, uncorrected deamination of methylated cytosine has a strong effect on the mutational rate.

Therefore, under antibiotic treatment, the emerged adaptive-resistant bacteria have higher mutation rate as consequence of the lower efficiency of DNA mismatch repair systems. This leads to two options: the bacteria can acquire lethal mutations that kill the cell or it can store advantageous mutations in order to reach a stable antibiotic resistance.

Furthermore, methylated bases are shown to be directly linked with mutation because they are mutational hotspots. A genomic study, performed by combining short reads with SMRT sequencing, revealed that methylated adenine (6 mA) are mutational hotspots in *Neisseria meningitidis* (Sater et al., 2015). The study of thousands of *E. coli* and *Salmonella spp*. genome assemblies revealed that there is mutation bias in methylation motifs (Cherry, 2018). As stated above, Dcm methylates the inner C residual of the motif 5′-CCWGG-3′, and the authors found that this C residual has a C to T mutation rate 8 fold higher than the other C residuals in the genome.

The authors also found a similar mutation bias for the motifs of the other RM methyltransferases. In another work, Cherry (2021) showed how 4 mC methylation caused by *Salmonella enterica* Type III RM system in the motif 5′-CAC4mCGT-3′ increases the transversion rate from the methylated cytosine to adenine by 500-fold.

## Methods used to investigate epigenetics in bacteria

Despite the importance of AdR and epigenetics in the emergence of antibiotic-resistant strains, these mechanisms remain poorly investigated. Among the reasons, the lack of suitable techniques to detect methylated bases is likely one of the main. Recently, the development of third-generation sequencing machines revolutionized the field allowing the precise identification of methylated bases. The main sequencing-based methods available for the analysis of DNA methylation are, namely, bisulfite sequencing, restriction enzymes–based mapping and third-generation sequencing (SMRT and Nanopore) (Supplementary Table S4).

Bisulfite sequencing technology (Beaulaurier et al., 2019) is able to detect 5 mC and 4 mC sites but is blind to m6A. Sodium bisulfite treatment converts the unmethylated cytosines of genomic DNA in uracil, while methylated cytosines remain intact. During library preparation uracil is converted to thymine; therefore, the resulting sequence can be compared with the sequence of untreated DNA to obtain the 5 mC positions. Four mC is detected by adding the TET (ten-eleven translocation) enzyme to the standard bisulfite sequencing protocol. This enzyme catalyzes the oxidation of 5 mC to 5-carboxylcytosine (5caC), which is read as thymine in the final sequence, remaining only 4 mCs as cytosine.

Restriction enzymes–based mapping is a sequencing protocol able to detect methylation of known motifs: it is based on the use of a couple of restriction enzymes that recognize a sequence if methylated or unmethylated. The resulting fragments will be analyzed with Next Generation Sequencing (NGS). This method is reliable but useful only if the restriction motif is known, and if methyl sensitive and insensitive specific restriction enzymes exist for that motif (Beaulaurier et al., 2019).

The recent advances in long-read sequencing technologies provided the means to directly investigate bacterial DNA methylation patterns. The emergence of third-generation sequencing machines allowed the detection of the N6-methyladenosine (m6A) and of the other modified bases of the DNA, along with the simultaneous detection of the nucleotide sequence. Two platforms are available for the detection of modified bases on a genomic scale (Payelleville and Brillard, 2021): Single Molecule, Real-Time (SMRT) sequencing (Eid et al., 2009) and the Oxford Nanopore technology (ONT). These approaches don't involve a replication step during the library preparation, reducing the time required before sequencing, and allowing the preservation of all the modifications on the genome.

Despite the great advantages provided by third-generation sequencing, at the state-of-the-art, some limits remain: (i) DNA has to be extracted from fresh samples; (ii) the high error rate of SMRT and Nanopore sequencing could include errors in the methylation analysis; and (iii) gold standard data analysis algorithms and software still lack.

### SMRT

Single Molecule, Real-Time (SMRT) sequencing can detect, with different sensitivity, all the main modifications of the DNA: it allows an optimal detection of 4 mc and m6A, but for 5 mC it requires additional steps, such as TET conversion or very deep sequencing coverage (Beaulaurier et al., 2019). In addition to that, SMRT sequencing also allows the detection of the phosphotioroation of the backbone of the DNA (Cao et al., 2014). The fundamental unity of this sequencing technology is composed by a zero-mode waveguide (ZMW), a small chamber in which the light converges, on which a DNA polymerase is immobilized. The template is a DNA double-stranded fragment, circularized and ligated with hairpin adaptors to each end (Eid et al., 2009), and it is anchored to the DNA polymerase at the bottom of the chamber. The enzyme proceeds multiple times on the template, adding fluorescently nucleotides (dNTP) complementary to the template base and labeled with four different fluorophores. Each base is added and immobilized for a short amount of time in the ZMW, its fluorescence pulse captured by a camera, and the set of all the pulses is used to construct the nucleotide sequence. There is an interval of time between every incorporating event that is called inter-pulse duration (IPD) and describes the polymerase kinetics. IPD is eventually modified by chemical modifications of the template DNA, so methylated bases or modification of the DNA backbone can be identified with the changes in polymerase kinetics (Ardui et al., 2018).

SMRT sequencing can be coupled with microarray techniques to link DNA methylation profiles with gene expression, allowing correlations between transcriptional gene levels and different methylation patterns in antibiotic resistance strains (Chen et al., 2018). This sequencing technique has shown to be useful to detect new methylation motifs (Blow et al., 2016), and it has been used to assess also the possible indirect correlations between the methylation status of a specific motif and the expression of a resistance gene (Spadar et al., 2021). It has been established that the absence of methylation in a motif downstream of a gene that is not transcribed may be a sign of a DNA conformation that prevents methyltransferase binding but also RNA polymerase binding. Thus, methylation analysis could provide information useful to identify distant regulatory regions or secondary DNA structure that can affect gene transcription (Spadar et al., 2021).

### Oxford Nanopore technologies

Similar to SMRT, Nanopore sequencing doesn't require an amplification step and allows the sequencing of very long reads (around 10 kb). This system works on a membrane filled with nanopore proteins, immersed in an electrolyte solution on which a voltage current is applied. When the single-strand DNA template passes through the nanopore protein, a nucleotide-specific electric alteration is produced and registered (Branton et al., 2008). The system can also detect methylated bases because they generate a distinctive current pattern, different from the unmethylated bases (Ciuffreda et al., 2021).

## Epigenetic analysis with third-generation sequencing technologies will represent a boost for antibiotic resistance studies

Bacterial epigenetics has a role in antibiotic resistance, and third-generation sequencing platforms are pivotal tools for the investigation of this phenomenon. Third-generation sequencing, and in particular SMRT, can be used for the detection of all the main epigenetic features of bacterial DNA, including the identification of methylated bases and modification of the DNA backbone. The use of these sequencing platforms could allow the discovery of novel mechanisms for the emergence of antibiotic-resistant strains. It is also possible that, in the future, the prediction of resistance phenotype based on bacterial genetics will also take into account epigenetic aspects. Furthermore, third-generation sequencing allows the comprehension of the mechanisms of genetic transfer, in order to monitor transfer and acquisition of resistance genes and mobile elements with resistance determinants. On the one hand, the combination of second- and third-generation sequencing platforms (short and long reads) allow us to investigate the link between methylation and mutation. On the other hand, third-generation sequencing data could be combined with RNA-seq or microarray experiment results to study the role of fast gene expression modulation in the emergence of antibiotic-resistant strains. Methylation analysis can be also useful to identify distant regulatory regions or secondary DNA structure that can prevent gene transcription and to correlate methylation patterns with different gene expression also in resistant strains. Exploring the epigenetic mechanism beyond the antibiotic resistance could also lead to the discovery of novel pharmacological targets, perhaps not subject to selective pressure and that could not lead to the development of new antibiotic resistance mechanisms.

## Conclusion

Epigenetic mechanisms in bacteria are still not fully understood, and the studies performed until now have only scratched the surface of the problem. This knowledge gap in such an important process can strongly limit our ability to understand the mechanisms beyond the emergence of antibiotic-resistant nosocomial pathogens.

Most of the experimental studies on DNA methylation have been carried out on *E. coli* by the knockout of methylation-associated genes, rather than focusing on the identification of methylated DNA bases. The study of the expression of genes involved in DNA methylation in *E. coli* during the early stages of exposure to antibiotics could help to highlight the pathways involved in this mechanism. Third-generation sequencing technologies directly collect methylation information during the DNA sequencing process, allowing us to investigate the relationship between methylation and mutation. In the future, improvements in standardization of methylation experiments and in sequencing data analysis will be pivotal to obtain more robust and comparable results.

## Author contributions

SPap performed the review of the literature, wrote the manuscript, and prepared the figures. AA, RN, and SPan performed the review of the literature. IB conceived the idea. FC conceived the idea and wrote the manuscript. All authors contributed to the article and approved the submitted version.

## Conflict of interest

The authors declare that the research was conducted in the absence of any commercial or financial relationships that could be construed as a potential conflict of interest.

## Publisher's note

All claims expressed in this article are solely those of the authors and do not necessarily represent those of their affiliated organizations, or those of the publisher, the editors and the reviewers. Any product that may be evaluated in this article, or claim that may be made by its manufacturer, is not guaranteed or endorsed by the publisher.
